# Adalimumab for severe psoriasis in Chilean paediatric patients^☆^^☆☆^

**DOI:** 10.1016/j.abd.2019.04.012

**Published:** 2019-12-30

**Authors:** Daniela Armijo Fernandez, Fernando Valenzuela, Gustavo Saint-Pierre Contreras, Andrea Cortés González

**Affiliations:** a Department of Dermatology, Hospital Clinico Universidad de Chile, Santiago, Chile; b Hospital San José de la Mariquina, Valdivia, Chile

Dear Editor,

Juvenile psoriasis is a chronic inflammatory skin disease affecting the 0.6–1.4% of paediatric patients.^1^ It is associated with several comorbidities including: psoriatic arthritis, psychiatric disorders, diabetes mellitus 1 and 2, hypertension, obesity, hyperlipidaemia and Crohn disease^2^, ^3^, ^4^; therefore, it is essential to establish an early and adequate treatment. Systemic therapeutic options in children are limited because of the adverse events, and approved biologic therapies in severe psoriasis at the moment are few. Adalimumab has been approved by the European Medicines Agency (EMA) for the treatment of severe plaque psoriasis in patients from 4 years of age, and recently a randomized, double-blind clinical trial has shown its safety and superiority compared to methotrexate.^5^ In Chile, there's no economic reimbursement for biological therapies and patients must pay on their own the full treatment, so the access to these therapies is limited due to the costs. The aim of our research is to present our experience with the use of adalimumab in paediatric patients with severe psoriasis.

Patients < 18 years old with severe psoriasis and lack of response to topical and/or systemic-non-biological therapies were selected to receive adalimumab 0.8 mg/kg subcutaneously at week 0, then every other week since week 1. They were followed-up for at least 40 weeks. Monthly clinical evaluations were performed; Psoriasis Area and Severity Index (PASI) and Physician Global Assessment 6 point scale (PGA) were assessed at all visits.

Four patients were selected, three were female. The median age at diagnosis of psoriasis was 11 years old and the mean duration of the disease before the use of adalimumab was 4.2 years. Two had positive family history of psoriasis but none of them were first-degree relatives. All of them had severe plaque psoriasis. Patient 4, presented severe genital psoriasis and HLA B27+ psoriatic arthritis with involvement of sacroiliac joints that had failed to respond to methotrexate. Patient 2 was overweight. No other metabolic, joint or psychiatric comorbidities were identified. Baseline disease characteristics included: median PASI 21.3, median PGA scores 4, and median Body Surface Area (BSA) 29%. At week 16, three out of four achieved PASI 75, and the median PGA score was 2. At week 40 the median PASI was 1.7, all of them achieved PASI 75 and two achieved PASI 90. All patients achieved PGA score 1–0 (Table 1, Table 2). Only mild adverse events were reported: upper respiratory tract infection (3 cases), lower respiratory tract infection (3 cases), and headache (1 case).

**Table 1:** Psoriasis area severity index, PASI.

	Baseline	Wk 16	Wk 40
Patient 1	18.3	3.1	1.7
Patient 2	12	3.1	1.8
Patient 3	40	0	2.1
Patient 4	12	1	0

**Table 2:** Physician global assessment, PGA.

	Baseline	Wk 16	Wk 40
Patient 1	3	2	1
Patient 2	3	2	1
Patient 3	5	0	1
Patient 4	3	0	0

We were unable to find any previous reports in Chilean paediatric patients with severe psoriasis treated with adalimumab. In our serie, the treatment with adalimumab 0.8 mg/kg in paediatric patients who had failed to previous non-biological therapies resulted in significant improvements in PASI 75 at week 16 that were maintained up to week 40.

Juvenile psoriasis has been associated with important comorbidities, and the estimated overall rate has been reported twice as high as in patients without psoriasis.^1^ Only one of the patients had psoriasis arthritis that had a good rate of response, improving joint symptoms and quality of life. Considering the comorbidities of juvenile psoriasis, the important detriment in quality of life and the recently published literature, adalimumab seems an effective and safety option for paediatric patients with severe psoriasis. However, as in our country there is no economic reimbursement for these therapies, the treatment of severe psoriasis that had failed to conventional management becomes challenging.

## Financial support

None declared.

## Authors’ contribution

Daniela Armijo Fernandez: Approval of the final version of the manuscript; elaboration and writing of the manuscript; collection, analysis, and interpretation of data; effective participation in research orientation; critical review of the literature; critical review of the manuscript.

Fernando Valenzuela: Approval of the final version of the manuscript; conception and planning of the study; collection, analysis, and interpretation of data; effective participation in research orientation; intellectual participation in the propaedeutic and/or therapeutic conduct of the studied cased; critical review of the manuscript.

Gustavo Saint-Pierre Contreras: Approval of the final version of the manuscript; elaboration and writing of the manuscript; collection, analysis, and interpretation of data; critical review of the literature.

Andrea Cortés González: Approval of the final version of the manuscript; collection, analysis, and interpretation of data; intellectual participation in the propaedeutic and/or therapeutic conduct of the studied cased; critical review of the manuscript.

## Conflicts of interest

None declared.
